# Upper Gastrointestinal Bleeding in Children: A Tertiary United Kingdom Children’s Hospital Experience

**DOI:** 10.3390/children4110095

**Published:** 2017-11-03

**Authors:** Omar Nasher, David Devadason, Richard J. Stewart

**Affiliations:** 1 Department of Paediatric Surgery, Queen’s Medical Centre, Nottingham University Hospitals NHS Trust, Derby Road, Nottingham NG7 2UH, UK; richard.stewart@nuh.nhs.uk; 2 Department of Paediatric Gastroenterology, Queen’s Medical Centre, Nottingham University Hospitals NHS Trust, Derby Road, Nottingham NG7 2UH, UK; david.devadason@nuh.nhs.uk

**Keywords:** upper gastrointestinal bleeding, general paediatrics, haematemesis, melaena, upper gastrointestinal endoscopy

## Abstract

The aim of this study was to review the aetiology, presentation and management of these patients with upper gastrointestinal bleeding (UGIB) at a tertiary children’s unit in the United Kingdom. This was a retrospective single-institution study on children (<16 years) who presented with acute UGIB over a period of 5 years using known International Classification of Diseases (ICD) codes. A total of 32 children (17 males, 15 females) were identified with a total median age at presentation of 5.5 years. The majority (24/32) of patients presented as an emergency. A total of 19/32 presented with isolated haematemesis, 8/32 with isolated melaena and 5/32 with a combination of melaena and haematemesis. On admission, the mean haemoglobin of patients who presented with isolated haematemesis was 11 g/dL, those with isolated melaena 9.3 g/dL and those with a combination 7.8 g/dL. Blood transfusion was required in 3/19 with haematemesis and 3/5 with haematemesis and melaena. A total of 19/32 underwent upper gastrointestinal endoscopy. Endoscopic findings were oesophageal varices (5/19) of which 4 required banding; bleeding gastric ulcer (1/19) requiring clips, haemospray and adrenaline; gastric vascular malformation (1/19) treated with Argon plasma coagulation therapy; duodenal ulcer (3/19) which required surgery in two cases; oesophagitis (5/19); and gastritis +/− duodenitis (3/19). A total of 13/32 patients did not undergo endoscopy and the presumed aetiology was a Mallory–Weiss tear (4/13); ingestion of foreign body (2/13); gastritis (3/13); viral illness (1/13); unknown (2/13). While UGIB is uncommon in children, the morbidity associated with it is very significant. Melaena, dropping haemoglobin, and requirement for a blood transfusion appear to be significant markers of an underlying cause of UGIB that requires therapeutic intervention. A multi-disciplinary team comprising gastroenterologists and surgeons is essential.

## 1. Introduction

Upper gastrointestinal bleeding (UGIB) is infrequent in children, with an estimated incidence of 1–2/10,000 per year [1], of which the majority are benign and self-limiting [2]. Significant UGIB is exceptional and consequently may pose a challenge to clinicians.

The aetiology of UGIB in children is heterogeneous and the exact cause varies with age, co-existing disease and geographical location [3,4]. In neonates, common causes include swallowed maternal blood and milk protein allergy, whereas in infants more common aetiologies are Mallory–Weiss tear and reflux oesophagitis. In older children and adolescents, significant causes of gastrointestinal haemorrhage include variceal bleeding secondary to portal hypertension, and peptic ulceration secondary to stress or mechanical trauma following foreign body ingestion [4]. 

This study reviews clinical practice with regard to aetiology, presentation and management of these patients at a tertiary children’s unit in the UK.

## 2. Methods

We performed a retrospective single institution study between May 2009 and May 2014 investigating the presentation, aetiology and management of children presenting with UGIB at Nottingham Children’s Hospital. 

International Classification of Diseases (ICD-10) codes for ‘Melaena’ (K921), ‘Haematemesis’ (K920), ‘Gastrointestinal haemorrhage’ (K922) and ‘Oesophageal varices with bleeding’ (I850), were utilised to search electronic discharge summaries for patients presenting during the study period. In addition, the electronic operating theatre database was searching to obtain information on patients who underwent endoscopy. Case notes were examined to record demographic details, mode of presentation and management. Patients who were 16 years of age or younger and presented with signs of UGIB such as melaena and/or haematemesis were included in this series. On the other hand, children who were older than 16 years of age or presented with signs of lower gastrointestinal bleeding such as bright red per rectum were excluded. Statistical analysis for any differences was performed using the χ^2^-test.

## 3. Results

During the study period we identified a total of 32 patients (17 males, 15 females) with a median age of 5.9 (± 5.5) years in males and 8.2 (± 6.0) years in females at presentation. 

A total of 24/32 (75%) of patients presented as an emergency via the Accident and Emergency department or having first presented to primary care. A further 6 (18.75%) were transferred from district general hospitals. Two were internal referrals of which one was an inpatient and one was referred having been seen in an outpatient clinic. Most children (24/32) were admitted under the paediatricians with the remainder under the care of the paediatric surgeons. 

A total of 19 (59.3%) patients presented with isolated haematemesis, 8 (25%) with isolated melaena and 5 (15.6%) with a combination of melaena and haematemesis. On admission, all but one had an initial full blood count performed. The mean haemoglobin (Hb) of patients who presented with isolated haematemesis was 11 g/dL (range: 5.1–14.3), those with isolated melaena had a mean Hb level of 9.3 g/dL (range: 5.5–13.4) and those presenting with a combination of haematemesis and melaena had a mean Hb of 7.8 g/dL (range: 5.1–10.6). 

**Figure 1:**
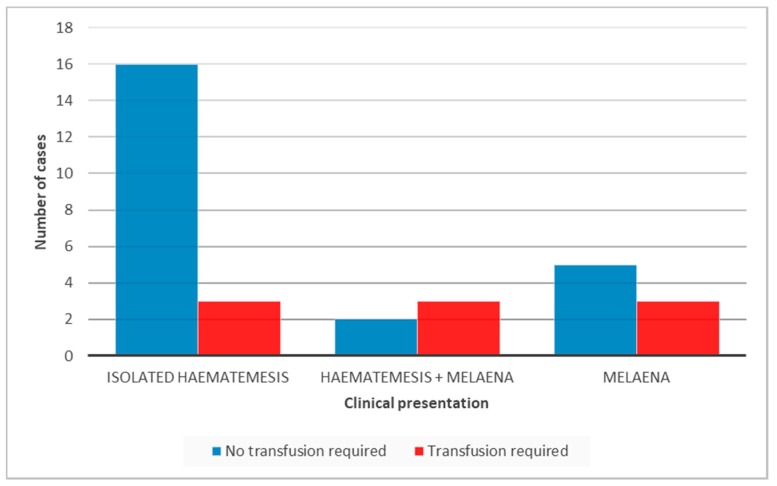
Clinical presentation and related blood transfusion requirement.

Patients with a significant drop in haemoglobin (<8 g/dL) and signs of haemodynamic instability were transfused. Consequently, a blood transfusion was required in 3/19 (15.7%) children with isolated haematemesis (Hb level of 5.1, 6.6 and 7.9 g/dL), and 3/5 (60%) with haematemesis as well as melaena (Hb level of 5.1, 6.4 and 6.6 g/dL). In addition, among those who needed a blood transfusion 3/8 (37.5%) presented with isolated melaena (Hb level of 5.5, 6.4 and 7.4 g/dL) (Figure 1). The difference in the transfusion requirement between the first group and the other two groups was not statistically significant.

Of the 32 patients, 19 (59.3%) underwent upper gastrointestinal endoscopy, 15 patients during the acute admission and 4 as a semi-elective procedure (Table 1). All 9 patients who required a blood transfusion proceeded to endoscopic assessment. At endoscopy, 5 patients were found to have oesophageal varices of whom 4 required banding of oesophageal varices. One had a bleeding gastric ulcer secondary to non-steroidal anti-inflammatory medication (NSAID) and was managed endoscopically with clips, haemospray and adrenaline; while another patient had a gastric vascular malformation that was treated with Argon plasma coagulation (APC) therapy. A total of 5 children had oesophagitis. Gastritis +/− duodenitis was documented in 3 patients at endoscopy, two of whom had evidence of Helicobacter pylori infection on biopsy, and the other one was on NSAID.

A duodenal ulcer was found in 3 patients. The first patient did not require any therapeutic intervention as it was healing. The second patient was found to have a non-bleeding duodenal ulcer at endoscopy but proceeded to laparoscopy because of significant clinical concerns and no further lesions were found. The third, a patient on the oncology ward undergoing chemotherapy, had an oesophago-gastro-duodenoscopy (OGD) where old blood was found in the stomach, and he proceeded to push enteroscopy, duodenotomy and defunctioning ileostomy with no definite cause identified for the bleeding. Then 48 h later, due to ongoing UGI haemorrhage, he underwent another laparotomy and was found to have a bleeding duodenal ulcer which was oversewn. Unfortunately, this patient had a further UGI haemorrhage leading to a fatal cardiac arrest.

All patients who had a blood transfusion proceeded to endoscopy. None of these patients had negative endoscopic findings. Endoscopic findings in the 9 patients who required a blood transfusion are listed in Table 2. Significantly, 7/9 patients required some form of therapeutic intervention including surgery. The absence of blood transfusion in our study meant that the chances of requiring therapeutic intervention was lower (*p*-value = 0.04). 

Three patients required further surgical intervention. A total of 2 were patients with duodenal ulcers (described above), including one with negative endoscopic findings. A third patient, following a negative endoscopy and laparoscopy, underwent a laparotomy and was found to have an intussusception secondary to intestinal metastases of osteosarcoma.

A total of 13/32 (40%) patients did not undergo endoscopic investigation. A total of 9/13 children presented with isolated haematemesis and the presumed aetiology based on history and clinical findings in those patients was a Mallory-Weiss tear (*n* = 4); ingestion of razor blades (*n* = 1); gastritis (*n* = 3, one of whom was on NSAID); and in one case the caused remained undetermined. A total of 4/13 patients had presenting features of melaena and the aetiology in those cases were known oesophageal varices (*n* = 1), transferred immediately to a quaternary centre for endoscopic management; ingestion of button battery which was spontaneously passed (*n* = 1); presumed viral illness (*n* = 1); and in one the cause remained undetermined, but this patient also underwent a Meckel’s scan which was negative.

None of these patients required a blood transfusion and remained haemodynamically stable during the course of their inpatient stay.

## 4. Discussion

In this study UGIB occurred in both males and females in an almost equal distribution. Similar to a study on 58 patients presenting with UGIB by Hassoon et al. [5]. Younger children were equally affected which is clinically significant due to their lower circulating blood volume. 

The majority of patients presented with isolated haematemesis (59%) whereas the others presented with either isolated melaena (25%) or a combination of both (16%). These findings reflect the results of previous studies on UGIB. In a cross-over study carried out in France, 96.6% of children presented with haematemesis, 14.1% with melaena and 2.8% with signs of haemorrhagic shock [1]. In another study conducted in the USA on children undergoing OGD to evaluate UGIB, the majority of patients presented with haematemesis (73.4%), whereas the remaining with melaena (20.8%) and coffee-ground emesis (5.7%) [3]. 

Melaena occurring in isolation or in combination with haematemesis predicts the need for transfusion in our series. The presence of melaena in the evaluation of a child, particularly in the younger children, is a hallmark of significant haemorrhage. The recognition and documentation of melaena could influence the clinical management, including nursing input these patients require. The presence of melaena independent of haematemesis indicates a significant UGIB, although in younger children an UGIB may manifest as altered blood per rectum. 

While the haemoglobin on admission may or may not reflect the true haemoglobin level, frequent monitoring of the full blood count should be adopted into practice. This is because a remarkable drop in haemoglobin identifies children with a significant bleeding who would benefit from early endoscopic intervention. In our study, the mean Hb of children who underwent endoscopy was 8.7 g/dL (range: 5.1–14.3). Those who were not subjected to endoscopy had a mean Hb level of 11.5 g/dL (range: 8.3–13.5). The Hb on admission was lower in children who presented with melaena, and even lower in children who presented with combination of haematemesis and melaena.

Our experience validates that if a transfusion is required, the likelihood of a significant endoscopic or laparoscopic finding of a cause of UGIB is high. In our study, the requirement of blood transfusion was a good predictor of endoscopic finding/intervention as all children who had a transfusion (9/32) also underwent upper gastrointestinal (UGI) endoscopy and were found to have significant findings (Table 2). 

The management approach to children presenting with UGIB is influenced by adult studies due to the lack of randomised controlled studies, systematic reviews or Cochrane reviews in children. In adult practice upper GI endoscopy is the gold standard for the management of upper gastrointestinal bleeding. However, early endoscopy within 24 h of presentation, remains a controversial topic [6]. In this series, the decision to carry out diagnostic and therapeutic endoscopy was based on clinical judgment and consequently 59% of patients underwent endoscopic investigation +/− intervention. Unlike adults, children frequently present with minimal UGIB and are managed expectantly as endoscopy requires a general anaesthetic. This is reflected in our series, where 13/32 (40%) of patients who presented with minimal UGIB did not undergo an upper GI endoscopy and, consequently, their diagnosis was presumptive. In our series, none of the 19 patients who had endoscopy had negative findings validating the decision-making process. 

Healthcare professionals involved in the management of these children need to distinguish significant UGIB, likely to require diagnostic +/− therapeutic endoscopy, from non-significant upper GI bleeds which are unlikely to benefit from endoscopic intervention.

This study demonstrates the heterogeneous nature and management of upper GI bleeding in children. The majority of cases were managed medically/endoscopically without the need of a laparotomy. In cases of severe UGI haemorrhage, a negative endoscopy does not necessarily rule out an underlying disorder, as demonstrated in our case series where two patients with inconclusive endoscopy went on to have exploratory surgery with findings to explain the cause of the gastrointestinal haemorrhage. 

There are no scoring systems in paediatrics although there are discussions about developing such a scoring system. Recently, Thompson et al., attempted to create a clinical scoring system to predict the need of urgent endoscopic intervention in children suffering from UGIB [7]. This is now awaiting prospective evaluation in clinical practice and subsequent validation. The numerous scoring systems used in the adult population, for example Rockall scoring system or Blatchford Addenbrookes scoring system, do not necessarily apply to children as many of the haematological and biochemical parameters are not relevant. This retrospective series shows that it may be possible to identify early children who will benefit from endoscopic intervention based on the parameters of drop in haemoglobin, requirement of blood transfusion, and melaena.

All UGIB can result in rapid deterioration requiring expertise to early identify those who will benefit from endoscopic intervention. Depending on the institution, access to out-of-hours endoscopy service may be limited. Given the infrequent occurrence of many of these conditions, paediatricians trained in endoscopy may not have sufficient expertise in dealing with therapeutic interventions in isolation. A 24/7 endoscopic service is essential, and each institution needs to have an agreement on which specialty should provide this service outside working hours. 

## 5. Conclusions

Healthcare professionals managing children need to be aware of the significant morbidity associated with UGIB, including the rapid deterioration in the clinical state of the child with UGIB and the potential for circulatory collapse.

The development of melaena, dropping haemoglobin, and requirement for a blood transfusion appear to be significant markers of an underlying cause of upper GI haemorrhage that may require therapeutic intervention. Incorporating these parameters in a validated scoring system would be beneficial.

Endoscopy units working with children need to develop protocols in conjunction with their multidisciplinary teams involving paediatric and adult gastroenterologists, paediatric surgeons and appropriately trained staff in therapeutic endoscopy. A low threshold is required to proceed to laparotomy when appropriate in the event of a negative endoscopy, and early planning and discussion of the roles within the multi-disciplinary team will be very useful particularly out of hours.

## Figures and Tables

**Table 1:** Endoscopic findings and therapeutic interventions.

Endoscopic Finding	Number of Patients (*N*)	Endoscopic or Other Therapeutic Intervention (*n/N*)
Varices	5	Banding (4/5)
Gastric ulcer	1	Endoclips, haemospray, adrenaline
Gastric vascular malformation	1	Argon plasma coagulation therapy
Gastritis/duodenitis	3	None
Oesophagitis	5	None
Duodenal ulcer	3	Laparoscopy (1), laparotomy (1), none (1)
No abnormality	1	Laparoscopy and laparotomy

**Table 2:** Haemoglobin (Hb) level at presentation and endoscopic findings/diagnosis in patients requiring blood transfusion.

Patient	Hb (g/dL)	Findings/Diagnosis	Endoscopic or Other Therapeutic Intervention
1	5.1	Gastritis/duodenitis/oesophagitis	None
2	7.9	Gastric ulcer	Endoclips, haemospray, adrenaline
3	6.4	Oesophageal varices	Banding
4	6.6	Oesophageal varices	Banding
5	5.5	Oesophageal varices	Banding
6	6.4	Oesophageal varices	None
7	6.6	Duodenal ulcer	Laparoscopy
8	5.1	Duodenal ulcer	Laparotomy
9	7.4	Secondary osteosarcoma deposits in small bowel	Laparoscopy and laparotomy
